# Postoperative function recovery in patients with endoprosthetic knee replacement for bone tumour: an observational study

**DOI:** 10.1186/s12891-018-2280-7

**Published:** 2018-10-02

**Authors:** Mattia Morri, Cristiana Forni, Riccardo Ruisi, Tiziana Giamboi, Fabrizio Giacomella, Davide Maria Donati, Maria Grazia Benedetti

**Affiliations:** 1 Servizio di Assistenza Infermieristica, Tecnica e della Riabilitazione, IRCCS Istituto Ortopedico Rizzoli, Via Pupilli 1, 40136 Bologna, Italy; 2 Clinica Ortopedica e Traumatologica III a prevalente indirizzo Oncologico, IRCCS Istituto Ortopedico Rizzoli, Bologna, Italy; 3 Servizio di Medicina Fisica e Riabilitativa, IRCCS Istituto Ortopedico Rizzoli, Bologna, Italy

**Keywords:** Bone tumors, Rehabilitation, Patient outcome assessment

## Abstract

**Background:**

The objective of this study is to describe the rehabilitative pathway of patients undergoing endoprosthetic knee replacement surgery, build reference values ​​of the functional results achieved, and identify possible prognostic factors.

**Methods:**

Prospective observational study. All patients undergoing resection and knee replacement surgery using a modular prosthesis following bone tumor resection were consecutively recruited over the last 2 years. The patients were followed for a period of 1 year, the result values ​​were collected at 3, 6 and 12 months.

**Results:**

In total, 30 patients were enrolled. The median age was 19 years with 33% of patients being female. Median values recorded for knee flexion, quadriceps strength, Toronto Extremity Salvage Score, Time Up and Go and Six Minutes Walking Test showed an improvement of 16, 25, 18, 48 and 38% from 3 to 12 months, respectively. The level and width of the resection were correlated with the mobility of the knee and the strength of the quadriceps.

**Conclusion:**

Patients undergoing knee replacement for bone tumors were able to achieve satisfactory functional outcomes from the first postoperative year. A specific assessment of outcomes can be conducted to facilitate the management of patient expectations. A very wide resection and interventions of the proximal tibia are risk factors for a poorer functional outcome.

## Background

Bone tumors are rare pathologies; the Italian Association of Tumor Registries (AIRTUM) reported a rate of 0.8 per 100.000 inhabitants for osteosarcoma. Malignant bone tumors represent approximately 5 to 6% of all tumors in young people [1, 2]. These occur more frequently in the metaphysis of long bones, especially at the knee and the proximal humerus [3]. With the improvement of diagnostic techniques, chemotherapy treatments and reconstructive techniques, most of these patients can be treated with a modular prosthesis after bone resection [4]; furthermore, the 5-year survival rate has improved from 20 to 85% [5, 6]. Because treatment and survival have improved, there is a need to manage residual impairment and disability in the medium and long term, bearing in mind that, being young, these patients will carry out very demanding motor activities. Indeed, several authors have underlined the achievement of good functional outcomes after surgery with modular prostheses [7–12], albeit with some physical limitations and a high rate of complications such as infections, mechanical failures and fractures of the implant [6]. In a study involving modular knee prostheses, Carty et al. [10] reported that at a mean follow up of 7.5 years (standard deviation, 5.1) the limitation of function and disability was correlated with the reduction of joint mobility and muscle strength. Moreover, balance was impaired, with greater difficulty in controlling posture in an upright position, particularly when such control was required with closed eyes. During walking, a lateral instability and asymmetry was reported [13]. To our knowledge, no rehabilitation protocols or specific care pathways are defined in the literature that attempt to achieve and improve these results. Bekkering (2012) et al. [8] reported their results at up to two-years’ follow-up with assessments at 3, 6, 9 12,18 and 24 months but rehabilitation methods in terms of intensity, type of exercises, and patient compliance with treatment are not well described. In addition, no predictive factors for recovery have been investigated. Only recently, Shehade et al. [14] attempted to describe and outline specific rehabilitation protocols for the different locations of the tumor. They concluded by advocating the use of standardized guidelines, as they can lead to an improvement in the final functional results. However, the paper does not report expected recovery times or whether good functional results can be achieved more quickly. The objective of the present study was to describe the rehabilitative pathway of patients undergoing knee replacement with modular prosthesis for bone tumour, as well as building reference values ​​of the functional results achieved in the consecutive rehabilitative phases (3, 6, and 12 months) to identify possible prognostic factors.

## Methods

### Study design: Prospective observational study

#### Participants

Between September 2014 and January 2016, all patients, of varying ages, undergoing resection and knee replacement surgery using a modular prosthesis for a primary musculoskeletal tumor, were consecutively recruited. Patients were surgically treated at the Oncological Orthopaedic Surgery Unit and followed for physical rehabilitation during the period of postoperative chemotherapy at the Chemotherapy Unit of an orthopedic university hospital. The only exclusion criterion was patient refusal to participate in the study. Patients who, during the follow-up, showed complications such as local tumor recurrence, implant infection and/or complications related to the administration of the antiblastic drug, which made it impossible to continue the rehabilitation process, were excluded from the study. Conversely, patients able to continue the study were re-evaluated at the next follow-up period. All patients provided written consent and the study protocol received formal approval from the Institute’s Ethics Committee (n. 0032914). The study variables included age, sex, diagnosis, resection level and length of resection. The patients were followed for the period of 1 year, with periodical assessment at 3, 6 and 12 months.

#### Outcome measure

During monitoring, an evaluation grid was used, based on the available literature and clinical experience. The aim of the grid was to obtain a summary of the patient’s main motor skills and it outlined 5 main result measures:

- Knee flexion/extension range of motion (ROM) of the knee [15], measured with a manual articular goniometer with the patient placed in the supine position. The patient was asked to flex and extend the knee as far as possible and then the physiotherapist applied further light pressure until the patient’s pain threshold was reached.
- The maximal strength of the quadriceps with the scale of the Medical Research Council [16]. In a sitting position, the patient was asked to extend the knee actively against the force of gravity and, where possible, against increasing pressure applied by the physiotherapist. This test was repeated for the healthy limb. The score ranged from 0, no muscular contraction, to 5, marked extension against manually applied pressure.
- The level of autonomy gained and perceived by the patient in everyday life according to the Toronto Extremity Salvage Score (TESS) [17] which is a self-administered patient questionnaire consisting of 30 items concerning the patient’s motor skills when performing daily life activities. Each item receives a minimum score of 1 to a maximum score of 5. The overall score is then expressed as a percentage; a greater percentage indicates greater autonomy.
- Motor performance, measured by Time Up and Go (TUG) [18]. This test was performed with the patient in a sitting position with hands on legs. The patient was asked to stand up, walk 3 m, turn around and come back. The test ended when the patient was sitting down again.
- Walking endurance, measured by the 6 minutes walking test (6mWT) [19]. The patient was asked to walk as far as possible in 6 min at a preferred speed.

#### Rehabilitation program

Patients were followed for rehabilitation in the Surgical Unit immediately after the intervention, and at each admission to the Chemotherapy Unit. Postoperative chemotherapy treatment consisted of a series of in-patient hospital admissions and the administration of 2–6-day continuous infusions for, in most cases, a total duration of about 6 months [20]. The rehabilitation program consisted of two daily sessions of therapy lasting at least 45 min each until patient discharge. The aim of the treatment was to guide the patient in the recovery process in order to minimize the disabling effects of surgery and to obtain the best possible recovery of residual abilities. The rehabilitation process was divided into two phases: in the initial phase, patients were prescribed a partial loading of the limb (15–20%) and then progressive loading during the second phase, up to complete weight bearing on the treated limb. The exact timeframe for increasing the load on the limb was decided by the orthopaedic surgeon according to the x-rays taken at 1, 3, and 6 months after surgery.

##### Partial weight-bearing phase (1st-2nd month)

The treatment was mainly aimed at recovering basic lower limb function such as walking, increasing knee mobility and strengthening the quadriceps. Passive and active knee flexion-extension and quadriceps strengthening exercises, with particular focus on the last degrees of extension were performed in a supine position with the use of a ball, following the indications in the literature [10]. In this initial phase proprioceptive exercises were performed in a sitting or standing position aimed at controlling the leg with the use of various aids such as a ball and rubber bands. For patients treated by proximal tibia resection, the use of a rigid knee brace was not recommended in the first 40 days, as it does not allow the mobilization of the knee. This period was necessary to obtain an adequate healing of the patellar tendon and entailed a delayed start to the knee mobilization exercises. During this initial phase, it was important to stimulate the patient’s proprioception of the treated limb by increasing the patient’s confidence with the prosthesis, in particular with the mechanical extension limitation.

##### Progressive weight-bearing phase (2nd-6th month)

**Fig. 1:**
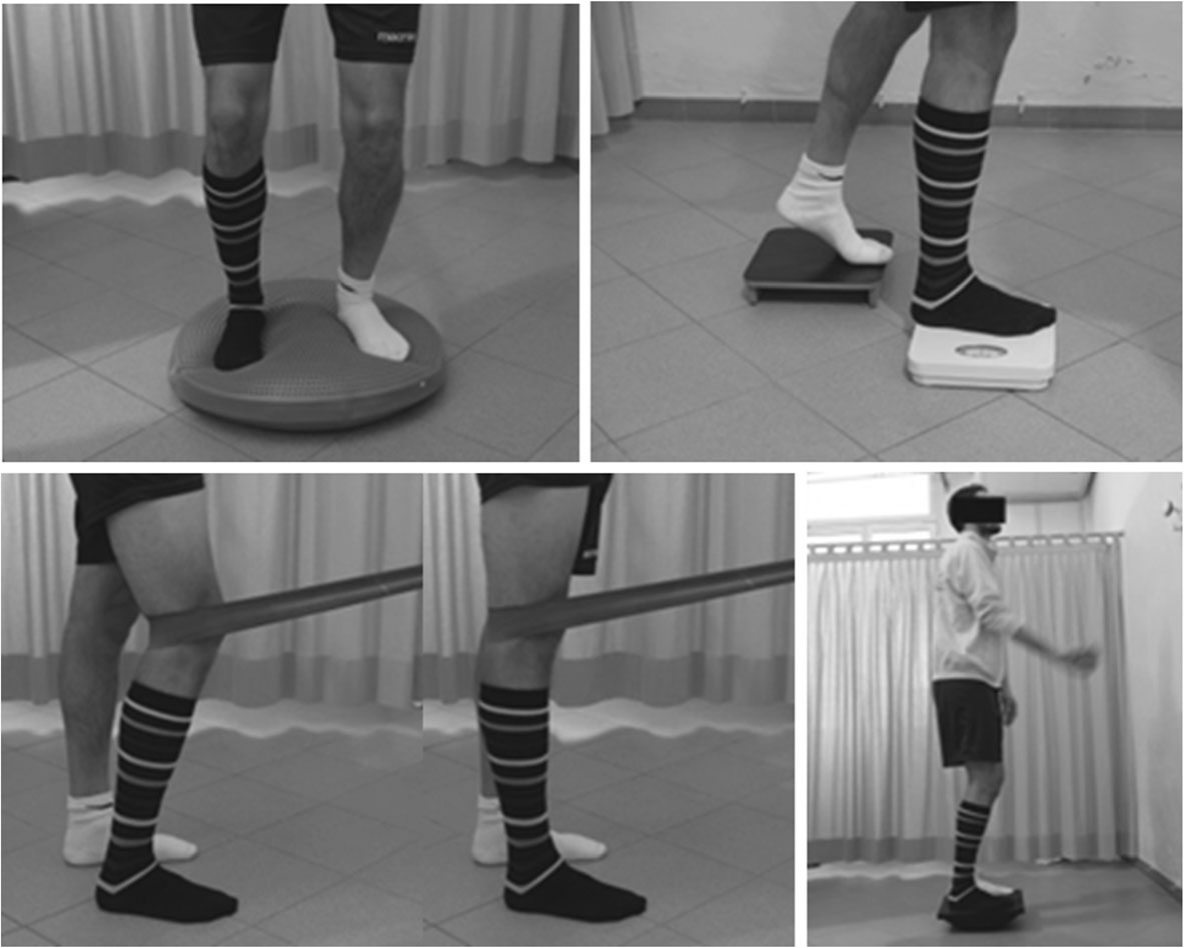
Exercises for patients with endoprosthetic knee replacement

From the time the patient was allowed 50% loading on the operated limb, the rehabilitative treatment included specific exercises in the standing position. Patients were asked to shift the load onto the limbs while maintaining a correct body alignment. Motor control of the treated knee might have been stimulated by a slight knee flexion or by the use of external resistance such as an elastic band. Exercises for two-leg standing were progressively made more challenging by modifying the support surface or using increasingly unstable surfaces and even the use of Freeman balance boards. To make the task even more challenging, closed-eye training or dual task exercises, such as throwing a ball and standing in an unstable position were introduced. Once full weight-bearing was allowed, the same exercises were carried out in the one-leg stance. Examples of the exercises are shown in Fig. 1 [21].

##### Use of the Wii-fit balance board

The Wii Balance Board was used as part of the physiotherapy treatment to test the shifting of load onto the lower limbs of a patient and assess the balance. The Balance Board is able to measure the distribution of body weight on the lower limbs of a player, according to changes in weight distribution under the sole of the foot. The player receives feedback from the exercise and the game they are playing. At the end of each game the console shows the score. The Wii Balance Board was used in one of the two scheduled rehab sessions every day throughout hospitalization for chemotherapy for at least 20 min using the exercises/games the console is equipped with. Exercises required patients to hold the center of gravity in an upright position and shift loads in the latero-medial, anterior-posterior and multidirectional direction, as described by Fung et al. [22]. The choice of games was left up to the patient and the lowest score achieved was the one recorded.

### Sample size

Bone tumors are rare [1]. Therefore, since it was not possible to define the number of cases necessary for the study as suggested by the usual statistical rules for observational studies, we arbitrarily decided to enroll all patients consecutively until a minimum number of 30 was reached.

### Statistical analysis

Statistical analysis was performed using IBM SPSS Statistics v. 21. Because the number of patients was small, all continuous data were expressed as median and the relative quartile (25th and 75th), categorical variables were expressed as proportions or percentages. Data concerning the outcomes measured at the various follow-ups were summarized in radar-type graphs. These graphs are a tool that can be used in clinical practice to compare the performance of new patients undergoing knee replacement with modular prosthesis. The Mann Whitney test was used to perform an analysis on subgroups of the main variables collected, such as age, sex, diagnosis, length of resection and level of resection and the functional outcome measured 1 year after surgery. In the absence of information in the literature concerning the length of resection, a cut-off of 20 cm was established from the observation of the data collected in the present study. *P* < 0.01 was considered statistically significant.

## Results

**Fig. 2:**
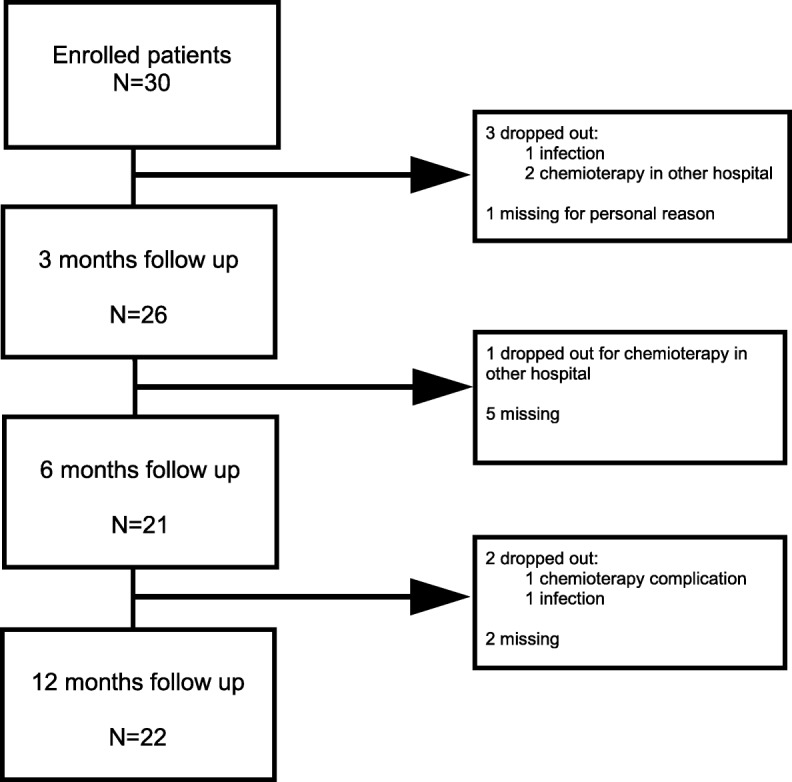
Enrolment process

**Fig. 3:**
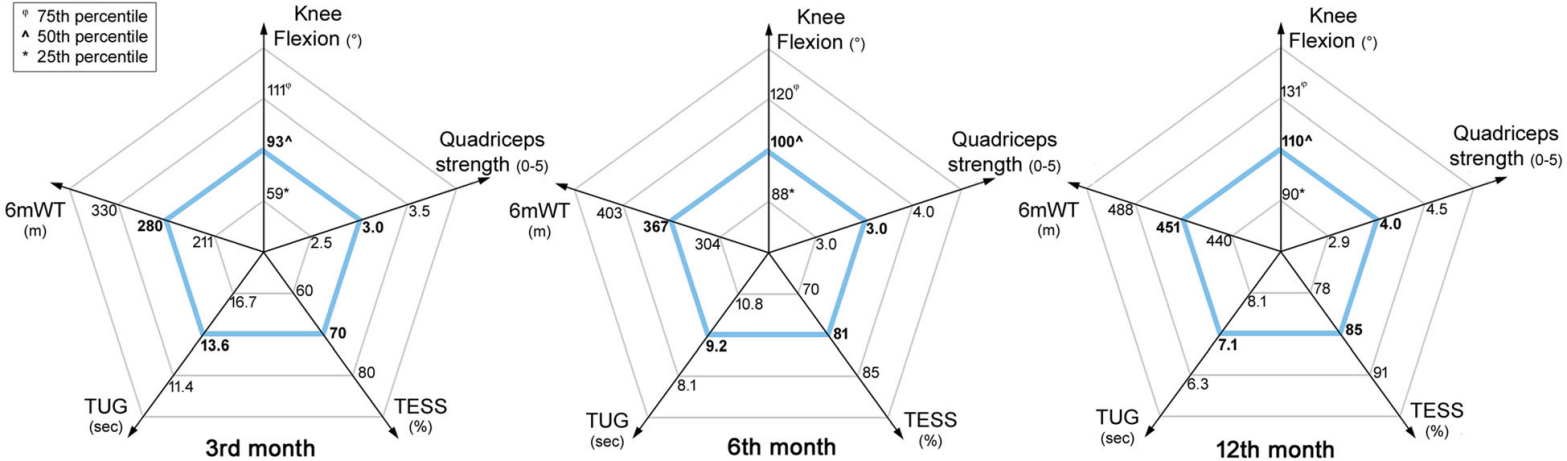
Description of patient recovery

In total, 30 patients were eligible for the study, and all were consecutively enrolled. At the 3rd, 6th and 12th month of follow up it was possible to evaluate 26, 21 and 22 patients, respectively. A description of the sample and its basic characteristics is shown in Table 1. The median age was 19 years with 33% of patients being female. At the 12-month follow up, 6 patients (20%) did not complete the 6-month rehabilitation program after surgery: 3 decided to continue the chemotherapy at another hospital, and 3 did not complete rehabilitation due to complications. The flow chart of patients leaving the study and patients evaluated at various times is shown in Fig. 2. The description of the recovery of patients over time is summarized in Fig. 3. The knee ROM extension level was not reported in the graphs or tables. No patient had limitations in this direction of movement. Data of the present study gathered at each follow up were summarized in a radar-type chart. All the functional measures taken into consideration showed an improvement in the three subsequent follow-ups. Median values recorded for knee flexion, quadriceps strength, TESS score, TUG and 6mWT showed an improvement of 16%, 25%, 18%, 48% and 38 between 3 and 12 months, respectively. Table 2 shows a comparison between the data found in the literature and the data of the present study. A median knee flexion of 110 degrees (41.3), a quadriceps strength of 4.0 (1.6), a TESS score of 85% (13.3), a TUG of 7.1 s (1.8) and 6mWT of 450 m (47.5) were in line with the values found in the literature [7–11]. The analysis of subgroups showed that the level of resection made a difference in the knee flexion range of motion, having a *p*-value of 0.04, and the length of resection made a difference in quadriceps strength, having a p-value of 0.03. The data set is summarized in Table 3.

**Table 1:** Patient characteristics, variables and functional results

Patient characteristics	*N* = 30
Median Age, years (min-max)	19 (9–66)
Female, n. (%)	10 (33.3)
Morphology, n. (%)	
Osteosarcoma	25 (83.3)
Ewing	5 (16.7)
Site of the tumor, n. (%)	
Femur	19 (63.3)
Tibia	11 (36.7)
Median resection of bone length, cm (min-max)	14.5 (11–30)
Median number of chemotherapy cycles, (min-max)	10.5 (5–15)
Complications, n (%)	9 (30%)
Infections	5
Mechanical failures	2
Intervention for pulmonary metastases	1
Chemo side effects	1

**Table 2:** Comparison of the results obtained between different studies

Authors (year)	N	Surgery	Follow-up (yr)	Flex (°)	Strength (0–5)	TESS (%)	TUG (sec)	6mWT (m)
Ginsberg, 2007 [7]	41	Limb sparing femur	4.29 (2.98)			86.4 (9.9)	6.6 (2.1)	
	24	Limb sparing tibia				88.1 (9.3)	6.0 (1.2)	
Henderson, 2012 [11]	12	Distal femur	4 (2–12)	98 (36)				
	3	Proximal tibia		105 (18)				
Carty, 2009 [10]	20	Limb salvage	7.5 (5.1)	125 (80–140)	4.15 (2–5)	86 (3.5)		
Bekkering, 2011 [9]	15	Knee endoprosthesis	2.3 (1.5)				7.2 (1.6)	471 (75)
Bekkering, 2012 [8]	4	Limb salvage and ablative	1			85 (2.4)		430 (18)
*Current Study*	*22*	*Knee endoprosthesis*	*1*	*110 (70–150)*	*4.0 (1.5–5.0)*	*85 (11.3)*	*7.1(1.8)*	*450 (47.5)*

**Table 3:** Multiple sub-group comparisons of the outcomes evaluated at 12 months

Variables		N	Flex °	Strenght	TESS	6mWT	TUG
Age	≤18y	9	110 (58)	3.4 (2.3)	87.5 (15.3)	450 (34)	7.5 (2.0)
	>18y	13	100 (28)	4 (1)	81.4 (12)	451 (108)	7.0 (1.8)
Sex	Men	17	110 (35)	4 (2)	83.6 (13.6)	450 (55)	7.4 (1.8)
	Women	5	90 (48)	4.5 (1.5)	87.5 (16.1)	468 (115)	6.9 (3.4)
Diagnosis	Osteosarcoma	18	110 (29)	3.75 (2)	85 (12.6)	449 (64)	7.5 (1.8)
	Ewing	4	112.5 (64)	4.5 (0.8)	85.3 (17.0)	459.5 (57)	6.3 (1.1)
Resection level	Femur	15	110 (40)	4 (1.5)	83.6 (13.1)	470 (80)	7.2 (1.9)
	Tibia	7	90 (40)	3.2 (1.5)	84.9 (14.1)	449 (25)	7.0 (1.6)
Resection amplitude	≤20 cm	16	105 (38)	4.5 (1)	91.9 (33.2)	449 (70)	7.3 (1.9)
	> 20 cm	6	115 (43)	3.5 (1.9)	82.7 (9.5)	469 (84)	6.8 (2.6)

## Discussion

Patients undergoing knee replacement with modular prostheses for musculoskeletal tumors can progressively achieve better functional levels during the first postoperative year. Knee resection entails a wide loss of bone and muscle structures resulting in a marked sensory-motor shock that has severe repercussions on the neuromotor control of the knee and balance, as documented by de Visser [13]. The choice of exercises in the patient’s recovery process is aimed at training the patient’s neuromotor control system from the initial postoperative phase. The results achieved by the population in the present study are in line with those of most previous studies [7–11] both in terms of the evaluation of disability and motor performance (Table 2). However, it should be noted that comparison with other studies must take into account the type of intervention patients were subjected to, and the timing of follow-up. Whereas the main studies report findings at a follow up of more than 2 years, patients analyzed in the present study were able to reach a similar functional outcome at 1 year of follow-up. Further studies are needed in order to understand if this result might be determined by the rehabilitation program implemented and if a prolonged treatment beyond a year can further improve outcomes. Benedetti et al. [23] highlighted the need to continue the rehabilitation of these patients for more than a year after surgery. In comparison with the paper by Bekkering [8], the only study to report the functional outcome data 1 year after the intervention, we observed the same level of autonomy achieved. In particular, we observed a TESS score of 85% and a similar distance walked during the 6mWT: 430 and 450 m, respectively. However, it should be noted that in Bekkering’s study [8] the functional data presented were not stratified by type of intervention; instead, patients treated with limb salvage and amputation were grouped together. It is important to underline the importance of iterative evaluation at 3–6-12 months to evaluate the progress of the patient’s recovery correctly by highlighting which skills are most deficient and which later on make functional recovery. In the field of oncology one of the tasks of rehabilitation is to guide the recovery of the patient and to address their expectations correctly [24]. The graphs presented in this study should be used as clinical tools able to provide indications on the progress of recovery, both for health care staff and patients. Sub-group analysis shows that when a resection of greater than 20 cm is made, a lower recovery of muscle strength with a median of 3.5 (IQR 1.9) is expected. Conversely, with resections below 20 cm, the median recovered force is 4.5 (IQR 1). This difference was not significant, having a more conservative *p*-value (*p* = 0.01). Regarding knee flexion, there is a difference between patients treated by distal femur resection (median 110° - IQR 40), compared to patients treated by tibia resection (median 90° - IQR 40). This difference may be the result of the immobilization period following the proximal tibia resection, necessary for the reconstructed patellar tendon to heal. Therefore, with regard to functional recovery in terms of force and mobility of the knee, two fundamental elements must be taken into consideration: the width of the resection greater than or less than 20 cm and the proximal tibia or distal femur resection.

### Limitation

The study has some limitations. First of all, the small sample size; bone tumors are rare and we collected all the patients admitted to our hospital over more than 1 year. Second, the arbitrary choice of 20 cm in the resection length. In the absence of information in the literature concerning the length of resection; that cut-off was established from the observation of the data collected in the present study. Finally, the lack of a control group.

## Conclusion

Patients undergoing knee replacement with modular prostheses for bone tumors are able to achieve satisfactory functional outcomes starting from the first postoperative year. A specific assessment of outcomes can be performed to facilitate the management of patient expectations and to help clinicians analyze the results achieved. A very wide resection and interventions of the proximal tibia might be risk factors for the functional outcome.

## Abbreviations

6mWT: Six minutes walking test
IQR: Interquartile range
ROM: Range of motion
TESS: Toronto Extremity Salvage Score
TUG: Time Up and Go
